# International society of sports nutrition position stand: caffeine and performance

**DOI:** 10.1186/1550-2783-7-5

**Published:** 2010-01-27

**Authors:** Erica R Goldstein, Tim Ziegenfuss, Doug Kalman, Richard Kreider, Bill Campbell, Colin Wilborn, Lem Taylor, Darryn Willoughby, Jeff Stout, B Sue Graves, Robert Wildman, John L Ivy, Marie Spano, Abbie E Smith, Jose Antonio

**Affiliations:** 1 Department of Exercise Science and Health Promotion, Florida Atlantic University, Boca Raton, FL 33431, USA; 2 The Center for Applied Health Sciences, Division of Sports Nutrition and Exercise Science, 3624 West Market Street, STE 104, Fairlawn, OH 44333, USA; 3 MRA Clinical Research, 6280 Sunset Drive #600, Miami, FL 33143, USA; 4 Department of Health and Kinesiology, Texas A & M University, College Station, TX 77843, USA; 5 University of South Florida, School of Physical Education and Exercise Science, Tampa, FL 33620, USA; 6 University of Mary Hardin-Baylor, Belton, TX 76513, USA; 7 Department of Health, Human Performance, and Recreation, Baylor University, Box 97313, Waco, TX 76798, USA; 8 Department of Health and Exercise Science, University of Oklahoma, Norman, OK 73019, USA; 9 Department of Human Nutrition, College of Human Ecology, Kansas State University, Manhattan, KS 66506, USA; 10 Department of Kinesiology and Health Education, the University of Texas, Austin, TX 78712, USA; 11 International Society of Sports Nutrition, 600 Pembrook Drive, Woodland Park, CO 80863, USA; 12 Nova Southeastern University, Fort Lauderdale-Davie, FL 33314, USA

## Abstract

Position Statement: The position of The Society regarding caffeine supplementation and sport performance is summarized by the following seven points: 1.) Caffeine is effective for enhancing sport performance in trained athletes when consumed in low-to-moderate dosages (~3-6 mg/kg) and overall does not result in further enhancement in performance when consumed in higher dosages (≥ 9 mg/kg). 2.) Caffeine exerts a greater ergogenic effect when consumed in an anhydrous state as compared to coffee. 3.) It has been shown that caffeine can enhance vigilance during bouts of extended exhaustive exercise, as well as periods of sustained sleep deprivation. 4.) Caffeine is ergogenic for sustained maximal endurance exercise, and has been shown to be highly effective for time-trial performance. 5.) Caffeine supplementation is beneficial for high-intensity exercise, including team sports such as soccer and rugby, both of which are categorized by intermittent activity within a period of prolonged duration. 6.) The literature is equivocal when considering the effects of caffeine supplementation on strength-power performance, and additional research in this area is warranted. 7.) The scientific literature does not support caffeine-induced diuresis during exercise, or any harmful change in fluid balance that would negatively affect performance.

## Introduction

Research on the physiological effects of caffeine in relation to human sport performance is extensive. In fact, investigations continue to emerge that serve to delineate and expand existing science. Caffeine research in specific areas of interest, such as endurance, strength, team sport, recovery, and hydration is vast and at times, conflicting. Therefore, the intention of this position statement is to summarize and highlight the scientific literature, and effectively guide researchers, practitioners, coaches, and athletes on the most suitable and efficient means to apply caffeine supplementation to mode of exercise, intensity, and duration.

## Caffeine and mechanism of action

To understand the effect of caffeine supplementation in its entirety it is necessary to discuss its chemical nature and how the compound is physiologically absorbed into the body. Caffeine is quickly absorbed through the gastrointestinal tract [1-3], and moves through cellular membranes with the same efficiency that it is absorbed and circulated to tissue [4,5]. Caffeine (1,3,7-trimethylxanthine) is metabolized by the liver and through enzymatic action results in three metabolites: paraxanthine, theophylline, and theobromine [1,6-8]. Elevated levels can appear in the bloodstream within 15-45 min of consumption, and peak concentrations are evident one hour post ingestion [1,3,9,10]. Due to its lipid solubility, caffeine also crosses the blood-brain barrier without difficulty [5,11]. Meanwhile, caffeine and its metabolites are excreted by the kidneys, with approximately 3-10% expelled from the body unaltered in urine [1,7,12]. Based on tissue uptake and urinary clearance circulating concentrations are decreased by 50-75% within 3-6 hours of consumption [3,13]. Thus, clearance from the bloodstream is analogous to the rate at which caffeine is absorbed and metabolized.

Multiple mechanisms have been proposed to explain the effects of caffeine supplementation on sport performance. However, several extensive reviews have stated that the most significant mechanism is that caffeine acts to compete with adenosine at its receptor sites [5,13,14]. In fact, in an exhaustive review of caffeine and sport performance, it was stated that "because caffeine crosses the membranes of nerve and muscle cells, its effects may be more neural than muscular. Even if caffeine's main effect is muscular, it may have more powerful effects at steps other than metabolism in the process of exciting and contracting the muscle [15]".

Clearly, one of caffeine's primary sites of action is the central nervous system (CNS). Moreover, theophylline and paraxanthine can also contribute to the pharmacological effect on the CNS through specific signaling pathways [5]. However, as noted above, rarely is there a single mechanism that fully explains the physiological effects of any one nutritional supplement. Because caffeine easily crosses the blood brain barrier as well as cellular membranes of all tissues in the body [15], it is exceedingly difficult to determine in which system in particular (i.e. nervous or skeletal muscle) caffeine has the greatest effect [15].

In addition to its impact on the CNS, caffeine can affect substrate utilization during exercise. In particular, research findings suggest that during exercise caffeine acts to decrease reliance on glycogen utilization and increase dependence on free fatty acid mobilization [16-19]. Essig and colleagues [19] reported a significant increase in intramuscular fat oxidation during leg ergometer cycling when subjects consumed caffeine at an approximate dose of 5 mg/kg. Additionally, Spriet et al. [18] demonstrated that following ingestion of a high dose of caffeine (9 mg/kg) net glycogenolysis was reduced at the beginning of exercise (cycling to exhaustion at 80% VO_2max_). Consequently, performance was significantly improved and results of this study [18] suggested an enhanced reliance on both intra- and extramuscular fat oxidation.

Another possible mechanism through which caffeine may improve endurance performance is by increasing the secretion of β-endorphins. Laurent et al. [20] demonstrated that when compared to the placebo group caffeine consumption (6 mg/kg) significantly increased plasma β-endorphin concentrations following two hours of cycling at 65% VO_2peak _and a subsequent bout of high intensity sprint activity. It has been established that plasma endorphin concentrations are enhanced during exercise and their analgesic properties may lead to a decrease in pain perception [21].

Research has also demonstrated that caffeine may result in alterations of neuromuscular function and/or skeletal muscular contraction [22,23]. For example, Kalmar and Cafarelli [22] indicated a moderate dose of caffeine (6 mg/kg) significantly enhanced both isometric leg extension strength as well as the time to fatigue during a submaximal isometric leg extension.

Caffeine consumption also promotes a significant thermogenic response. In fact, caffeine consumption at a dose of 100 mg resulted in a significant thermogenic effect despite the fact that subjects in that particular investigation had a habitual caffeine intake of 100-200 mg per day [24]. The increase in energy expenditure subsequent to caffeine ingestion had not returned to baseline 3 hours post-consumption.

Overall, the findings of research studies involving caffeine supplementation and physical performance indicate a combined effect on both the central and peripheral systems. Therefore, it is possible that caffeine acts on the central nervous system as an adenosine antagonist, but may also have an effect on substrate metabolism and neuromuscular function. Research in all areas of caffeine supplementation continues to emerge and it is necessary to understand that as a supplement, caffeine has wide ranging physiological effects on the body that may or may not result in an enhancement in performance. Caffeine supplementation can improve sport performance but this is dependent upon various factors including, but not limited to, the condition of the athlete, exercise (i.e. mode, intensity, duration) and dose of caffeine.

## Caffeine and Cognitive Performance

Caffeine has been shown to enhance several different modes of exercise performance including endurance [8,16,25-28], high-intensity team sport activity [29-34], and strength-power performance [30,35]. Additionally, the use of caffeine has also been studied for its contribution to special force operations, which routinely require military personnel to undergo periods of sustained vigilance and wakefulness. In a series of investigations, McLellan et al. [36-38] examined the effects of caffeine in special force military units who routinely undergo training and real life operations in sleep deprived conditions, where alertness and diligent observation are crucial to assignment.

In the McLellan et al. investigations [36-38], soldiers performed a series of tasks over several days, where opportunities for sleep were exceedingly diminished. Experimental challenges included a 4 or 6.3 km run, as well as tests for marksmanship, observation and reconnaissance, and psychomotor vigilance [36-38]. During periods of sustained wakefulness, subjects were provided caffeine in the range of 600-800 mg, and in the form of chewing gum. The caffeine supplement was consumed in this manner as it has been shown to be more readily absorbed, than if it was provided within a pill based on the proximity to the buccal tissue [39]. In all three studies [36-38], vigilance was either maintained or enhanced for caffeine conditions in comparison to placebo. Additionally, physical performance measures such as run times and completion of an obstacle course were also improved by the effects of caffeine consumption [36,38].

Lieberman et al. [40] examined the effects of caffeine on cognitive performance during sleep deprivation in U.S. Navy Seals [40]. However, in this investigation [40] the participants were randomly assigned varying doses of caffeine in capsule form delivering either 100, 200, or 300 mg. In a manner similar to previous investigations, participants received either the caffeine or placebo treatment and one hour post consumption performed a battery of assessments related to vigilance, reaction time, working memory, and motor learning and memory. In addition, the participants were evaluated at eight hours post consumption to assess duration of treatment effect in parallel to the half-life of caffeine, in a manner similar to a study conducted by Bell et al. [41].

As to be expected, caffeine had the most significant effect on tasks related to alertness [40]. However, results were also significant for assessments related to vigilance and choice reaction time for those participants who received the caffeine treatment. Of particular importance are the post-hoc results for the 200 and 300 mg doses. Specifically, there was no statistical advantage for consuming 300, as opposed to 200 mg [40]. In other words, those trainees who received the 300 mg (~4 mg/kg) dose did not perform significantly better than those participants who received 200 mg (~2.5 mg/kg). Meanwhile, a 200 mg dose did result in significant improvements in performance, as compared to 100 mg. In fact, it was evident from post-hoc results that 100 mg was at no point statistically different or more advantageous for performance than a placebo. These studies [36-38,40] demonstrate the effects of caffeine on vigilance and reaction time in a sleep deprived state, in a distinct and highly trained population. These findings suggest that the general population may benefit from similar effects of caffeine, but at moderate dosages in somewhat similar conditions where sleep is limited.

An additional outcome of the Lieberman et al. [40] study is the fact that caffeine continued to enhance performance in terms of repeated acquisition (assessment of motor learning and short-term memory) and Profile of Mood States fatigue eight hours following consumption. These results are in agreement with Bell et al. [41], where aerobic capacity was assessed 1, 3, and 6 hours following caffeine consumption (6 mg/kg). Caffeine had a positive effect on performance for participants classified as users(≥ 300 mg/d) and nonusers (≤ 50 mg/d); however, nonusers had a treatment effect at 6 hours post-consumption, which was not the case for users - this group only had a significant increase in performance at 1 and 3 hours post- consumption. Taken together, results of these studies [40,41] provide some indication, as well as application for the general consumer and athlete. Specifically, while caffeine is said to have a half-life of 2.5-10 hours [42], it is possible performance-enhancing effects may extend beyond that time point as individual response and habituation among consumers varies greatly.

Finally, it was suggested by Lieberman and colleagues [40] that the performance-enhancing effects of caffeine supplementation on motor learning and short-term memory may be related to an increased ability to sustain concentration, as opposed to an actual effect on working memory. Lieberman et al. [40] attributed the effects of caffeine to actions on the central nervous system, specifically the supplement's ability to modulate inhibitory actions, especially those of adenosine. In fact, it was suggested that because caffeine has the ability to act as an antagonist to adenosine, alterations in arousal would explain the compound's discriminatory effect on behaviors relating vigilance, fatigue and alertness [40].

Recently, it was also suggested that caffeine can positively affect both cognitive and endurance performance [25]. Trained cyclists, who were moderate caffeine consumers (approximated at 170 mg/d) participated in three experimental trials consisting of 150 min of cycling at 60% VO_2max _followed by five minutes of rest and then a ride to exhaustion at 75% VO_2max_. On three separate days, subjects consumed a commercially available performance bar that contained either 44.9 g of carbohydrates and 100 mg of caffeine, non-caffeinated-carbohydrate and isocaloric, or flavored water. Results from a repeated series of cognitive function tests favored the caffeine treatment in that subjects performed significantly faster during both the Stroop and Rapid Visual Information Processing Task following 140 min of submaximal cycling as well as after a ride to exhaustion. In addition, participant time increased for the ride to exhaustion on the caffeine treatment, as compared to both the non-caffeinated bar and flavored water [25].

Overall, the literature examining the effects of caffeine on anaerobic exercise is equivocal, with some studies reporting a benefit [29-32,43,44] and others suggesting that caffeine provides no significant advantage [45,46]. As with all sports nutrition research, results can vary depending on the protocol used, and in particular, the training status of the athlete as well as intensity and duration of exercise. For example, Crowe et al. [47] examined the effects of caffeine at a dose of 6 mg/kg on cognitive parameters in recreationally active team sport individuals, who performed two maximal 60-second bouts of cycling on an air-braked cycle ergometer. In this investigation [47], untrained, moderately habituated (80-200 mg/d) participants completed three trials (caffeine, placebo, control) and underwent cognitive assessments prior to consumption of each treatment, post-ingestion at approximately 72-90 min, and immediately following exercise. Cognitive testing consisted of simple visual reaction time and number recall tests. Participants performed two 60-second maximal cycle tests interspersed by three min of passive rest. The results were in contrast to other studies that investigated cognitive parameters and the use of caffeine [25,36-38,40] in that caffeine had no significant impact on reaction time or number recall, and there was no additional benefit for measurements of power. In fact, in this study [47], the caffeine treatment resulted in significantly slower times to reach peak power in the second bout of maximal cycling.

Elsewhere, Foskett and colleagues [48] investigated the potential benefits of caffeine on cognitive parameters and intermittent sprint activity and determined that a moderate dose (6 mg/kg) of caffeine improved a soccer player's ball passing accuracy and control, thereby attributing the increase in accuracy to an enhancement of fine motor skills.

Based on some of the research cited above, it appears that caffeine is an effective ergogenic aid for individuals either involved in special force military units or who may routinely undergo stress including, but not limited to, extended periods of sleep deprivation. Caffeine in these conditions has been shown to enhance cognitive parameters of concentration and alertness. It has been shown that caffeine may also benefit endurance athletes both physically and cognitively. However, the research is conflicting when extrapolating the benefits of caffeine to cognition and shorter bouts of high-intensity exercise. A discussion will follow examining the effects of caffeine and high-intensity exercise in trained and non-trained individuals, which may partially explain a difference in the literature as it pertains to short-term high-intensity exercise.

## Caffeine and Carbohydrate

An extensive body of research has provided compelling evidence to support the theory that caffeine's primary ergogenic mode of action is on the CNS. However, caffeine may also be ergogenic in nature by enhancing lipolysis and decreasing reliance on glycogen utilization. In 1979, Ivy et al. [16] published an investigation that supported the latter concept [16]. Trained cyclists were subjected to two hours of isokinetic cycling and received three treatments on separate occasions: caffeine, glucose polymer, and placebo. Caffeine was consumed in an absolute dose of 500 mg, 250 mg one hour prior to cycling and the remainder in divided doses beginning 15 min prior to onset of exercise. Results indicated a significant advantage in work produced following caffeine consumption. Specifically, work produced was 7.4% greater over control and 5.3% greater than the glucose polymer treatment. Midway into two hours of cycling, fat oxidation was significantly increased above that of the control and glucose trials. Fat oxidation was maintained during the last hour of exercise and it was suggested this substrate utilization was in part responsible for the increased work production. Moreover, following caffeine consumption and a two-hour bout of isokinetic cycling, plasma free fatty acid (FFA) levels were 30% greater than those for placebo.

Results of the Ivy et al. [16] study, as well as others [18,49], provide a persuasive argument for the use of caffeine as a means to increase work production by way of increased fat oxidation. However, Ivy et al. [16] suggested caffeine also had an effect on the CNS. Specifically, when subjects consumed caffeine, they began the exercise bout at a higher intensity, but perceived this effort to be no different than when they ingested the placebo and glucose conditions. Furthermore, Ivy et al. [16] also suggested participants were able to perform at this increased work rate due to a greater ability to rely on fat metabolism.

In a study performed by Jackman et al. [50] subjects consumed either caffeine at a dose of 6 mg/kg or placebo and performed high-intensity work with both the power output and total work done held constant. In total, subjects performed approximately 4-6 min of high intensity work (2-min bouts of cycling interspersed with 6 min of rest and a final ride to voluntary exhaustion). Results indicated an increase in plasma epinephrine for the caffeine treatment, which is consistent with other caffeine supplementation studies [8,29,46,51,52]. Even though epinephrine promotes glycogenolysis, the data from this study demonstrated an increase in both muscle lactate and plasma epinephrine without a subsequent affect on net muscle glycogenolysis following the first two bouts of controlled maximal cycling. Epinephrine can up-regulate lipolysis in adipocytes as well as glycogenolysis in muscle and liver; therefore, a direct relationship between increases in the hormone and enhanced substrate catabolism is somewhat ambiguous. Greer et al. [53] reported in 2000 that theophylline is more potent than caffeine as an adenosine antagonist. Whereas adenosine can act to inhibit lipolysis in vivo [54], theophylline consumption at 4.5 mg/kg resulted in increased blood glycerol levels, even more so than caffeine at 6 mg/kg and placebo. Indeed, it is possible that both theophylline and caffeine act to regulate substrate metabolism via mechanisms other than those that are catecholamine-induced [53].

Graham and Spriet [8] examined varying doses of caffeine consumption at 3, 6, and 9 mg/kg on endurance capacity (run to exhaustion at 85% VO_2max_). Results from this study demonstrated an enhancement in performance, but only with the 3 and 6 mg/kg dose. Concurrently, the 6 and 9 mg/kg dosages were the only measured quantities that resulted in increased plasma epinephrine levels, with significant increases in glycerol and free fatty acids measured only at the 9 mg/kg dose. Therefore, results of this investigation present quite a paradox in that a low dose of caffeine (3 mg/kg) was adequate for enhancing performance, but did not lead to increased levels of epinephrine or subsequent effect of free fatty acid mobilization.

Hulston and Jeukendrup [55] published data that indicated caffeine at 5.3 mg/kg co-ingested with a 6.4% glucose solution had no significant effect on increasing plasma FFA levels or glycerol concentrations, nor did it substantially enhance rates of whole-body fat oxidation during endurance exercise even though performance was significantly improved with the caffeine + glucose solution [55]. Therefore, the results of some research studies lend substantiation to the premise that caffeine may act to increase performance by altering substrate utilization [16,18], while results of additional investigations serve to suggest other mechanisms of action [50,56,57].

Carbohydrate consumption during exercise can decrease the body's dependence on endogenous carbohydrate stores and lead to enhanced endurance performance [58,59]. Therefore, it is beneficial to determine an optimal method of enhancing rates of exogenous carbohydrate delivery and oxidation. Exogenous carbohydrate delivery is determined by various factors including, but not limited to, the rate of gastric emptying and intestinal absorption [58]. However, it has been suggested that during exercise intestinal absorption seems to have the greatest influence on the rate of exogenous carbohydrate oxidation [58,60].

In 1987 Sasaki et al. [61] reported that in trained distance runners 100 g sucrose in combination with approximately 400 mg (~6 mg/kg) of caffeine had no additive effect on endurance performance, when compared to consumption of either substrate alone. In addition, Jacobson et al. [62] reported that caffeine (6 mg/kg) combined with carbohydrate (2.6 g/kg), had no significant enhancement on exercise performance or substrate utilization in trained cyclists.

However, Yeo et al. [63] reported that during the final 30 min of a 2-hr steady state bout of cycling (64% V02_max_) a 5.8% glucose solution (48 g/hr), in addition to 5 mg/kg of caffeine, significantly enhanced exogenous carbohydrate oxidation (~26% higher than glucose alone). It was suggested by these authors [63] and others [64] that this was the result of enhanced intestinal glucose absorption.

Finally, Hulston et al. [55] found that a 6.4% glucose solution in addition to a moderate dose of caffeine (5.3 mg/kg) significantly enhanced time trial performance in trained cyclists. The caffeine-glucose solution improved performance by 9% when compared to placebo and 4.6% in comparison to glucose. However, it was also reported that caffeine consumption had no affect on exogenous carbohydrate oxidation [55]. In addition, Kovacs et al. [56] demonstrated that after consuming caffeine at a dose of either 225 mg or 320 mg in combination with a carbohydrate-electrolyte solution participants were able to perform significantly faster during a time trial protocol. In contrast, Desbrow and colleagues [65] found a low dose of caffeine (1.5 and 3 mg/kg), in addition to glucose consumption every 20 min had no significant affect on time trial performance nor did caffeine in combination with glucose, affect maximal exogenous carbohydrate oxidation [65].

Strategies that may enhance exogenous carbohydrate absorption and oxidation during exercise are clearly defined in the literature [58-60]. The combined effect of caffeine and exogenous carbohydrate intake during endurance exercise is less understood. Therefore, future research should continue to investigate this potential ergogenic effect, as well as any corresponding physiological mechanisms.

### Caffeine, carbohydrate, and recovery

Recently, the combination of caffeine and carbohydrate has been examined as a potential means to enhance recovery by increasing the rate of glycogen synthesis post exercise. In 2004, Battram et al. [66] demonstrated that following carbohydrate depleting exercise, exogenous carbohydrate and caffeine supplementation did not hinder either proglycogen (small particles) or macroglycogen (large, acid soluble) production. It was postulated that the fractions respond differently to the recovery phase of exercise and thus glycogen resynthesis. Prior to, as well as during exhaustive exercise, subjects consumed in divided doses a total of 6 mg/kg of either caffeine or placebo in capsule form. Following exercise and throughout the 5-hr recovery period subjects consumed in total 375 g of exogenous carbohydrate. Muscle biopsies and blood samples revealed caffeine ingestion did not obstruct proglycogen or macroglycogen resynthesis following exhaustive, glycogen depleting exercise [66]. It is imperative to recognize that each person may respond differently to supplements and compounds containing caffeine. An individual at rest, and even sedentary in nature, is likely to have a different response compared to a trained, conditioned athlete, or physically active person. According to the data presented by Battram et al. [66], caffeine supplementation followed by exogenous carbohydrate in the recovery phase did not negatively impact glycogen resynthesis.

In a more recent study, Pedersen et al. [67] investigated the role of caffeine plus carbohydrate as a post-exercise method for enhancing glycogen synthesis. Following an overnight fast, trained cyclists and triathletes performed exhaustive exercise on a cycle ergometer at 70% VO_2peak_. In a crossover manner, subjects consumed 4 g CHO/kg (gels, sports bars, carbohydrate-containing drinks) and on another day 4 g CHO/kg in the same form, in addition to caffeine at 8 mg/kg, which was added to a carbohydrate-containing sports drink and consumed in two divided doses. Following a 4-hr recovery period, results were definitive in that glycogen resynthesis was increased by 66% for the carbohydrate-caffeine treatment, as compared to the carbohydrate-only condition [67].

The data presented in these studies [66,67] indicate that caffeine is not detrimental to glycogen repletion, and in combination with exogenous carbohydrate may actually act to enhance synthesis in the recovery phase of exercise. From a practical standpoint, however, it should be considered that most athletes or recreationally trained individuals would choose to supplement with caffeine prior to competition for the purpose of enhancing performance. Moreover, clearance of caffeine in the bloodstream occurs between 3 and 6 hours, and may extend beyond that time point depending on the individual. Therefore, caffeine consumption pre- and post-exercise would have to be precisely timed so as not to interrupt sleep patterns of the athlete, which in itself could negatively affect overall recovery.

## Caffeine: Form, Dose, and Endurance Exercise

### Caffeinated coffee, anhydrous caffeine and endurance exercise

Various methods of caffeine supplementation have been explored and results have provided considerable insight into appropriate form and dosage of the compound. One of the most acknowledged studies, published by Graham et al. [26] demonstrated a range of effects when caffeine (at 4.45 mg/kg) was consumed in varying forms. In their study, aerobically conditioned runners performed five treadmill runs to exhaustion at approximately 85% VO_2max _after receiving one of the following treatments 60 minutes prior: caffeine capsules plus water, regular coffee, decaffeinated coffee, decaffeinated coffee plus caffeine in capsule form, and placebo. Caffeine in capsule form significantly increased work capacity allowing them to run an additional 2-3 km [26], as compared to the four other treatments.

It was also proposed by Graham and colleagues [26] that perhaps other indistinguishable compounds within coffee rendered caffeine less effective than when consumed in anhydrous form. This suggestion was supported by de Paulis et al. [68] in a 2002 publication which indicated derivatives of chlorogenic acids are produced from the roasting process of coffee. In turn, these derivatives may have the potential for altering the affects of caffeine as an adenosine antagonist, possibly reducing the drug's ability to diminish the inhibitory action of adenosine [68].

As such, McLellan and Bell [27] examined whether a morning cup of coffee just prior to anhydrous caffeine supplementation would have any negative impact on the compound's ergogenic effect. Subjects were physically active and considered to be moderate-to-high daily consumers of caffeine. In a crossover design consisting of six separate testing days, rides to exhaustion were performed at approximately 80% VO_2max_. Subjects consumed one cup of coffee with a caffeine dosage that was approximately 1.0 mg/kg, and 30 min later ingested either of the following six conditions: decaffeinated coffee + placebo capsules; decaffeinated coffee + caffeine capsules at 5 mg/kg, coffee at 1.1 mg/kg + caffeine capsules at 5 mg/kg, coffee + caffeine capsules at 3 mg/kg, coffee + caffeine capsules at 7 mg/kg, water + caffeine capsules at 5 mg/kg. The results indicated caffeine supplementation significantly increased exercise time to exhaustion regardless of whether caffeine in anhydrous form was consumed after a cup of regular or decaffeinated coffee [27]. Taken together the available research suggests that caffeine supplemented in capsule form in a range of 3 to 7 mg/kg provided an average increase in performance of 24% over placebo [27]. While caffeine supplemented from a cup of coffee might be less effective than when consumed in anhydrous form, coffee consumption prior to anhydrous supplementation does not interfere with the ergogenic effect provided from low to moderate dosages.

### Caffeinated coffee, decaffeinated coffee, and endurance exercise

Wiles et al. [69] examined the effect of 3 g of coffee, which contained approximately 150-200 mg of caffeine, on treadmill running time. This form and dose was used to mimic the real life habits of an athlete prior to competition. Subjects performed a 1500-m treadmill time trial. Ten subjects with a VO_2max _of 63.9-88.1 ml/kg/min also completed a second protocol designed to simulate a "finishing burst" of approximately 400 m. In addition, six subjects also completed a third protocol to investigate the effect of caffeinated coffee on sustained high-intensity exercise. Results indicated a 4.2 s faster run time for the caffeinated coffee treatment, as compared to decaffeinated coffee. For the "final burst" simulation, all 10 subjects achieved significantly faster run speeds following ingestion of caffeinated coffee. Finally, during the sustained high-intensity effort, eight of ten subjects had increased VO_2 _values [69].

In a more recent publication, Demura et al. [70] examined the effect of coffee, which contained a moderate dose of caffeine at 6 mg/kg, on submaximal cycling. Subjects consumed either caffeinated or decaffeinated coffee 60 min prior to exercise. The only significant finding was a decreased RPE for the caffeinated coffee as compared to the decaffeinated treatment [70].

Coffee contains multiple biologically active compounds; however, it is unknown if these compounds are of benefit to human performance [71]. However, it is apparent that consuming an anhydrous form of caffeine, as compared to coffee, prior to athletic competition would be more advantageous for enhancing sport performance. Nevertheless, the form of supplementation is not the only factor to consider as appropriate dosage is also a necessary variable.

### Low, moderate, and high dosages of anhydrous caffeine and endurance exercise

Pasman and colleagues [28] examined the effect of varying quantities of caffeine on endurance performance. Nine aerobically trained cyclists performed six rides to exhaustion at approximately 80% maximal power output. Subjects consumed four treatments on separate occasions: placebo, 5, 9, and 13 mg/kg of caffeine in capsule form. Results were conclusive in that all three caffeine treatments significantly increased endurance performance as compared to placebo. Moreover, there was no statistical difference between caffeine trials. Therefore, increases in performance were comparable for both the moderate dose of 5 mg/kg as well as the high dose of 13 mg/kg [28]. The average increase in performance time was 27% for all three caffeine treatments [28], and are analogous to the U.S. Navy SEAL training study published by Lieberman et al [40]. Results from that paper indicated no statistical advantage for consuming an absolute dose of 300 mg, as opposed to 200 mg. However, the 200 mg dose did result in significant improvements in performance, as compared to 100 mg, and 100 mg was at no point statistically different or more advantageous for performance than placebo [40].

As previously discussed, Graham and Spriet [8], examined the effects of varying quantities of caffeine on metabolism and endurance exercise and reported a significant increase in performance for a low (3 mg/kg) and moderate dose (6 mg/kg) of caffeine but not for 9 mg/kg. In response to why a low and moderate dose of caffeine significantly enhanced performance, as compared to a high dose, Graham and Spriet [8] suggested that, "On the basis of subjective reports of some subjects it would appear that at that high dose the caffeine may have stimulated the central nervous system to the point at which the usually positive ergogenic responses were overridden". This is a very pertinent issue in that with all sports nutrition great individuality exists between athletes, such as level of training, habituation to caffeine, and mode of exercise. Therefore, these variables should be considered when incorporating caffeine supplementation into an athlete's training program.

### Anhydrous caffeine and endurance exercise

In an earlier study published by Graham and Spriet [52], seven elite runners performed a total of four trials, two cycling to exhaustion and two running to exhaustion at approximately 85% VO_2max_. Times for running and cycling were both significantly improved, running increased from ~49 min for placebo to 71 min for 9 mg/kg of caffeine, cycling increased from ~39 min for placebo to ~59 min for 9 mg/kg of caffeine [52].

Results were comparable in a separate 1992 Spriet et al. publication [18]. In a crossover design eight subjects consumed both a placebo and caffeine treatment at 9 mg/kg and 60 minutes later cycled to exhaustion at ~80% VO_2max_. Once again, following caffeine supplementation times to exhaustion were significantly increased. Results indicated subjects were able to cycle for 96 min during the caffeine trial, as compared to 75 min for placebo [18].

Recently McNaughton et al. [72] reported the effects of a moderate dose of caffeine (6 mg/kg) on 1-hour time trial performance. This investigation is unique to the research because, while continuous, the protocol also included a number of hill simulations to best represent the maximal work undertaken by a cyclist during daily training. The caffeine condition resulted in the cyclists riding significantly further during the hour-long time trial, as compared to placebo and control. In fact, time trial performance was improved 4-5% by the caffeine treatment over the other two treatments [72].

The use of caffeine in anhydrous form, as compared to a cup of caffeinated coffee, seems to be of greater benefit for the purpose of enhancing endurance performance. In addition, a low-to-moderate dose of caffeine between 3 and 6 mg/kg appears to be sufficient for enhancing performance in a maximal sustained endurance effort.

## Caffeine: High-Intensity and Team Sport Exercise

It is evident that caffeine supplementation provides an ergogenic response for sustained aerobic efforts in moderate-to-highly trained endurance athletes. The research is more varied, however, when pertaining to bursts of high-intensity maximal efforts. Collomp et al. [46] reported results for a group of untrained subjects, who participated in only 2-3 hours per week of non-specific sport activity. In a fasted state, and in a crossover design, subjects consumed caffeine at a dose of 5 mg/kg as well as a placebo condition, and performed a 30-second Wingate test. Compared to a placebo, caffeine did not result in any significant increase in performance for peak power or total work performed [46]. These results are in agreement with Greer and colleagues [45], where in addition to a lack of performance enhancement with caffeine supplementation (6 mg/kg), subjects classified as non-trained experienced a decline in power, as compared to placebo, during the last two of four Wingate bouts [45]. As previously stated, Crowe et al. [47] reported significantly slower times to reach peak power in the second of two bouts of 60-s maximal cycling. Subjects in that study were untrained in a specific sport and consumed caffeine at a dose of 6 mg/kg [47]. Finally, Lorino et al. [47] examined the effects of caffeine at 6 mg/kg on athletic agility and the Wingate test. Results were conclusive in that non-trained males did not significantly perform better for either the pro-agility run or 30-s Wingate test [73]. In contrast, a study published by Woolf et al. [30] demonstrated that participants who were conditioned athletes achieved greater peak power during the Wingate after consuming caffeine at a moderate dose of 5 mg/kg [30]. It is exceedingly apparent that caffeine is not effective for non-trained individuals participating in high-intensity exercise. This may be due to the high variability in performance that is typical for untrained subjects.

Results, however, are strikingly different for highly-trained athletes consuming moderate doses of caffeine. Collomp et al. [46] examined the use of 250 mg of caffeine (4.3 mg/kg) in trained and untrained swimmers. Swimmers participated in two maximal 100 m freestyle swims; significant increases in swim velocity were only recorded for the trained swimmers. Similar results were reported by MacIntosh and Wright [74] in a study that examined the effects of caffeine in trained swimmers, but the caffeine treatment was provided at a higher dose (6 mg/kg) and the protocol involved a 1,500-meter swim. Results indicated a significant improvement in swim times for those subjects who consumed caffeine, as compared to placebo. Moreover, time was measured at 500-m splits, which resulted in significantly faster times for each of the three splits for the caffeine condition [74]. As suggested by Collomp et al., [29] it is possible that specific physiologic adaptations present in highly trained anaerobic athletes, such as enhanced regulation of acid-base balance (i.e., intracellular buffering of H^+^), is intrinsic for caffeine to exert an ergogenic effect [29].

Participants in a study published by Woolf et al. [30] were highly trained anaerobic athletes, and results of that investigation demonstrated a significant increase in peak power with a moderate dose of caffeine (5 mg/kg) as compared to placebo [30]. Wiles et al. [44] reported a 3.1% improvement in performance time for a 1-kilometer time trial (71.1s for caffeine; 73.4s for placebo) at a caffeine dose of 5 mg/kg, and results also included a significant increase in both mean and peak power [44]. Wiles et al. [44] indicated that subjects in the study reported regular interval sprint training, which may support the theory that caffeine is most beneficial in trained athletes who possess physiological adaptations to specific high-intensity training [44].

A recent study published by Glaister et al. [31] examined a 5 mg/kg dose of caffeine on sprint interval performance. Subjects were defined as physically active trained men and performed 12 × 30 m sprints at 35 s intervals. Results indicated a significant improvement in sprint time for the first three sprints, with a consequential increase in fatigue for the caffeine condition [31]. The authors suggested that the increase in fatigue was due to the enhanced ergogenic response of the caffeine in the beginning stages of the protocol and, therefore, was not meant to be interpreted as a potential negative response to the supplement [31].

Bruce et al. [32] tested two doses of caffeine (6 mg/kg, 9 mg/kg) on 2000 m rowing performance in competitively trained oarsmen. Results of the study revealed an increase in performance for both time trial completion and average power output for caffeine, as compared to placebo (500 mg glucose). Time trial completion improved by 1.3% for caffeine intake at 6 mg/kg. The 9 mg/kg dose did not result in additional increases in performance. The average of the 6 and 9 mg/kg caffeine treatments was 1.2% faster as compared to placebo [32]. Anderson and colleagues [75] tested these same doses of caffeine in competitively trained oarswomen, who also performed a 2,000-m row. In women, the higher dose of 9 mg/kg of caffeine resulted in a significant improvement in time by 1.3%, with performance enhancement most evident in the first 500 m of the row [75].

Team sport performance, such as soccer or field hockey, involves a period of prolonged duration with intermittent bouts of high-intensity playing time. As such, Stuart et al. [33] examined the effects of a moderate dose of caffeine (of 6 mg/kg) in well-trained amateur union rugby players. Subjects participated in circuits that were designed to simulate the actions of a rugby player, which included sprinting and ball passing, and each activity took an average 3-14 seconds to complete. In total, the circuits were designed to represent the time it takes to complete two halves of a game, with a 10 min rest period. Results demonstrated a 10% improvement in ball-passing accuracy [33]. An improvement in ball passing accuracy is applicable to a real-life setting as it is necessary to pass the ball both rapidly and accurately under high-pressure conditions [33]. In addition, throughout the duration of the protocol, those subjects on the caffeine condition successfully passed the ball 90% of the time as compared to 83% for placebo [33]. This study [33] was the first to show an improvement in a team sport skill-related task as it relates to caffeine supplementation. Results of this study [33] also indicated that for the caffeine condition subjects were able to maintain sprint times at the end of the circuit, relative to the beginning of the protocol.

Schneiker et al. [34] also examined the effects of caffeine supplementation on repeated sprint ability common to sports such as soccer and field hockey. Ten male recreationally competitive team sport athletes took part in an intermittent-sprint test lasting approximately 80 minutes in duration. Results of the study indicated a caffeine dose of 6 mg/kg was successful in inducing more total sprint work, as compared to placebo. Specifically, total sprint work was 8.5% greater in the first half and 7.6% greater in the second respectively [34].

Based on the research presented [29,30,33,34,74], it is apparent that moderate caffeine supplementation in the range of 4-6 mg/kg can be advantageous to either short term or intermittent/prolonged duration high-intensity performance, but only in trained athletes. The training and conditioning of these athletes may result in specific physiologic adaptations which, in combination with caffeine supplementation, may lead to performance enhancement, or the variability in performance of untrained subjects may mask the effect of the caffeine.

## Caffeine: Strength- Power Performance

In the area of caffeine supplementation, strength research is still emerging and results of published studies are varied. As previously mentioned, Woolf and colleagues [30] examined the effects of 5 mg/kg of caffeine in highly conditioned team sport male athletes. The protocol consisted of a leg press, chest press, and Wingate. The leg and chest press consisted of repetitions to failure (i.e., muscular endurance) and all exercises were separated by 60 seconds of rest. Results indicated a significant increase in performance for the chest press and peak power on the Wingate, but no statistically significant advantage was reported for the leg press, average power, minimum power, or percent decrement [30].

Beck et al. [35] examined the acute effects of caffeine supplementation on strength, muscular endurance, and anaerobic capacity. Resistance trained males consumed caffeine (201 mg, equivalent to 2.1-3.0 mg/kg) one hour prior to testing. Subjects were tested for upper (bench press) and lower body (bilateral leg extension) strength, as well as muscular endurance, which consisted of repetitions to exhaustion at 80% of individual 1RM. Participants were also tested for peak and mean power by performing two Wingate tests separated by four minutes of rest (pedaling against zero resistance). A low dose of 2.1-3.0 mg/kg of caffeine was effective for increasing bench press 1RM (2.1 kg = 2.1%). Significant changes in performance enhancement were not found for lower body strength in either the 1RM or muscular endurance [35].

Results of the Beck et al. [35] investigation are in contrast to a recent publication by Astorino et al. [76] in which twenty-two resistance-trained men were supplemented with 6 mg/kg of caffeine and tested on the bench press and leg press [76]. Findings from Astorino and colleagues [76] revealed no significant increase for those subjects supplemented with caffeine for either bench or leg press 1RM. Astorino et al. [76] did report a nonsignificant increase in repetitions and weight lifted at 60% 1RM for both the bench and leg press [76]; however, the intensity differed between the two studies. The Beck et al. design included a 2.1-3.0 mg/kg dose of caffeine and repetitions to failure at 80% of individual 1RM, whereas subjects in the Astorino et al. investigation consumed 6 mg/kg and performed repetitions to failure at 60% of individual 1RM. Indeed it is possible that the degree of intensity between the two protocols could in some way be a resulting factor in the outcome of the two studies.

Consequently, Woolf and colleagues [77] reported no significant increase in bench press performance in collegiate football athletes who consumed a moderate dose of caffeine (5 mg/kg) 60 min prior to testing. Participants in this investigation [77] were considered non-habituated to caffeine and consumed much less than 50 mg per day.

Research on the effects of caffeine in strength-power sports or activities, while varied in results and design, suggest that supplementation may help trained strength and power athletes. Therefore, future research should examine the effect of caffeine habituation and supplementation on strength and/or high-intensity short duration exercise. Of particular interest, is the lack of significant finding for lower body strength as compared to upper body performance.

## Caffeine and Women

Research investigations that have examined the role of caffeine supplementation in endurance, high-intensity, or strength-trained women is scant, especially in comparison to publications that have investigated these dynamics in men. As previously indicated, Anderson and colleagues [75] examined the effect of both a moderate and high dose (6 and 9 mg/kg) of caffeine in competitively trained oarswomen. Results from a 2,000 m row performance signified the higher dose of caffeine (9 mg/kg) resulted in a significant improvement in time by 1.3%, with performance enhancement most evident in the first 500 m of the row. In addition, no significant increase in performance was reported for the lower dose or placebo; but the 6 mg/kg dose did result in a non-significant 0.7% improvement [75].

Motl et al. [78] examined the effects of a 5 and 10 mg/kg dose of caffeine on leg muscle pain during cycling to exhaustion at 60% VO_2peak_. Subjects were of average physical fitness and designated as non-habituated (consumed less than 100 mg/day of caffeine). Based on a leg muscle pain ratings scale, it was found that caffeine at both the 5 and 10 mg/kg dose significantly decreased leg muscle pain ratings during exercise [78]. Moreover, there was no statistically significant difference between the 5 and 10 mg dose [78]. The lack of a dose-dependent effect is in line with previously published investigations [8,28,32,40].

In two different publications, Ahrens and colleagues [79,80] examined the effects of caffeine supplementation on aerobic exercise in women. In one study [79] recreationally active women not habituated to caffeine participated in moderately-paced (3.5 mph) treadmill walking for eight minutes. In a double-blind manner, subjects randomly consumed caffeine mixed with water at either 3 or 6 mg/kg of body weight. The initial design included a 9 mg/kg dose, but during the first lab visit seven of ten subjects who received that treatment experienced profuse sweating, body tremors, dizziness, and vomiting. Results for the caffeine treatment at 6 mg/kg, as compared to 3 mg/kg and placebo, yielded a significant increase in energy expenditure at seven additional calories per 30 minutes of moderate walking [79]. From a research standpoint the increase in VO_2 _(0.67 ml/kg/min, equivalent to an increase in rate of energy expenditure of 0.23 kcal/min) is significant; however, in a practical setting it seems slightly less considerable. Finally, no significant results were reported for caffeine and aerobic dance bench stepping [80].

Goldstein and colleagues [81] examined the effects of caffeine on strength and muscular endurance in resistance-trained females. Similar to results reported by Beck et al. [35] it was found that a moderate dose of caffeine (6 mg/kg) significantly enhanced upper body strength (bench press 1RM). Women in this study were required to bench press 70% of individual body weight to be identified as resistance trained [81].

The research pertaining exclusively to women is somewhat limited and exceptionally varied. Publications range from examining caffeine and competitive oarswomen [75] to others that have investigated recreationally active individuals performing moderate-intensity aerobic exercise [79,80]. Taken together, these results indicate that a moderate dose of caffeine may be effective for increasing performance in both trained and moderately active females. Additional research is needed at all levels of sport to determine if caffeine is indeed effective for enhancing performance in women, either in a competitive or recreationally active setting.

## Caffeine, Habituation, and Performance

It is standard procedure for a research protocol to account for the daily caffeine intake of all subjects included within a particular study. The purpose of accounting for this type of dietary information is to determine if caffeine consumption a.) has an effect on performance and b.) if this outcome is different between a person who does or does not consume caffeine on a regular basis. In fact, as previously discussed in this paper Bell and colleagues [41] examined the effect of a moderate dose of caffeine on persons identified as users (≥ 300 mg/d) and nonusers (≤ 50 mg/d). Results demonstrated an enhancement in performance for both groups; however, the treatment effect lasted approximately three hours longer for those persons identified as nonusers [41].

Dodd et al. [82] identified caffeine habituation between subjects in a similar manner to Bell and colleagues [41] and reported no statistical difference between groups for VO_2max _(subjects participated in a graded exercise protocol). The only reported differences, such as ventilation and heart rate, were at rest for those persons not habituated to caffeine [82]. Van Soeren et al. [83] also reported no significant changes between users and nonusers of caffeine, other than an increase in plasma epinephrine during exercise for persons not habituated to caffeine, as compared to placebo. Finally, it was suggested by Wiles et al. [69] that daily caffeine consumption among subjects did not have an effect on the performance outcomes of that particular study, which examined the effects of 3 g of coffee containing approximately 150-200 mg of caffeine, on treadmill running time.

What may be important to consider is how caffeine affects users and nonusers individually. For example, Astorino and colleagues [76] examined the effects of 6 mg/kg of caffeine on bench press one-repetition maximum. Thirteen of 22 subjects in that investigation described feelings of greater energy, elevated heart rate, restlessness, and tremor. It should also be noted that these feelings were enhanced in participants who consumed little caffeine on a daily basis [76]. It would seem the important factor to consider is the individual habits of the athlete and how caffeine supplementation would affect their personal ability to perform. In terms of practical application, it is the responsibility of the coach and/or athlete to determine what dose of caffeine, if any, is suitable for competition.

## Caffeine and Hydration

It has been widely suggested that caffeine consumption induces an acute state of dehydration. However, consuming caffeine at rest and during exercise presents two entirely different scenarios. Specifically, studies examining the effects of caffeine-induced diuresis at rest can and should not be applied to athletic performance. To begin, a study published in 1928 by Eddy & Downs [84] examined the possible role of caffeine induced dehydration but included an *n *of only 3. In a review publication on caffeine and fluid balance, it was suggested by Maughan and Griffin [85] that "hydration status of the individual at the time of caffeine ingestion may also affect the response, but this has not been controlled in many of the published studies".

Despite the unfounded, but accepted, notion that caffeine ingestion may negatively alter fluid balance during exercise, Falk and colleagues [86] found no differences in total water loss or sweat rate following consumption of a 7.5 mg/kg dose of caffeine (5 mg/kg 2 hr prior to exercise, 2.5 mg/kg 30 min prior) and treadmill walking with a 22-kg backpack (intensity of ~70-75% VO_2max_). The authors did caution that exercise was carried out in a thermoneutral environment and additional research is warranted to determine effects in a more stressful environmental condition [86].

Wemple et al. [87] investigated the effects of a caffeinated versus non-caffeinated electrolyte solution drink at rest and during 180 min of moderate-intensity cycling at 60% VO_2max_. In total, 8.7 mg/kg of caffeine was consumed in divided doses. Results indicated a significant increase in urine volume for caffeine at rest, but there was no significant difference in fluid balance for caffeine during exercise [87]. These results are noteworthy, because according to a review published by Armstrong [88], several research studies published between 1970 and 1990 reported outcome measures, such as loss of water and electrolytes, based on urine samples taken at rest and within 2-8 hours of supplementation [88].

Kovacs and colleagues [56] published similar results in a 1998 study that examined time trial performance and caffeine consumption in various dosages added to a carbohydrate-electrolyte solution (CES). In total, subjects consumed each carbohydrate-electrolyte drink with the addition of 150 mg, 225 mg, and 320 mg of caffeine. In regard to performance, subjects achieved significantly faster times following ingestion of both the CES 225 mg and CES 320 mg dosages, as compared to placebo and CES without addition of caffeine [56]. Finally, Kovacs et al. [56] found no statistical difference in urine volume either before or after cycling. It should also be mentioned the authors reported wide-ranging post-exercise urinary caffeine concentrations within subjects, which could possibly be explained by inter-individual variation in caffeine liver metabolism [56]. Grandjean et al. [89] collected urine samples over a 24-hr period and found at rest there was no significant change in urine output at rest when consuming water or varying doses of caffeine in the range of 114 mg/d-253 mg/d (1.4 mg/kg - 3.13 mg/kg).

An interesting study published by Fiala and colleagues [90] investigated rehydration with the use of caffeinated and caffeine-free Coca-Cola^®^. In a double-blind crossover manner, and in a field setting with moderate heat conditions, subjects participated in three, twice daily, 2-hr practices. Athletes consumed water during exercise, and on separate occasions, either of the Coca-Cola^© ^treatments post-exercise. In total, subjects consumed ~7 cans/d or ~741 mg/d of caffeine. As a result, no statistical differences were found for measures such as heart rate, rectal temperatures, change in plasma volume, or sweat rate [90]. It should be noted, however, the authors also reported a negative change in urine color for the mornings of Day 1 and 3, which was a possible indication of an altered hydration status; although, it was not evident at any other time point during the experiment. Therefore, Fiala et al. [90] suggested future research should continue to investigate the effects of rehydrating with caffeine over several consecutive days.

Roti et al. [91] examined the effects of chronic caffeine supplementation followed by an exercise heat tolerance test (EHT). The study included 59 young, active males. All subjects consumed 3 mg/kg of caffeine for six days, and during days 7-12 subjects were divided into three groups and ingested 0, 3, or 6 mg/kg of caffeine. The EHT consisted of walking on a treadmill at 1.56 m/s at a 5% grade. Results were conclusive in that sweat rates were not statistically different between groups, and chronic supplementation of 3 and 6 mg/kg of caffeine did not negatively affect fluid-electrolyte balance, thermoregulation, and thus performance.^91^.

Millard-Stafford and colleagues [92] published results from a study that examined the effects of exercise in warm and humid conditions when consuming a caffeinated sports drink. No significant differences were found for any of the three treatments: placebo (artificially flavored water), 6% carbohydrate-electrolyte, and 7% carbohydrate-electrolyte plus B vitamins 3, 6, and 12 in addition to 46 mg/L carnitine, 1.92 g/L taurine, and 195 mg/L caffeine for sweat rate, urine output, or percent fluid retained during exercise [92]. In fact, a significant increase in exercise intensity was reported for the final 15 min (an all out portion of the exercise bout) for the caffeine + carbohydrate and electrolyte beverage, but not for the carbohydrate + electrolyte drink, or placebo. In conclusion, no significant differences in blood volume were present for any of the three treatments; therefore, caffeine did not adversely affect hydration and thus performance of long duration in highly trained endurance athletes [92].

Finally, Del Coso and colleagues [93] examined the effects of a moderate dose of caffeine in combination with sustained cycling at 60% VO_2max_. Seven endurance-trained males consumed each of the following conditions during 120 min of exercise: no rehydration, water, carbohydrate-electrolytes solution, and each of these three treatments with the addition of caffeine at 6 mg/kg in capsule form. Results were conclusive, and indicated caffeine alone at 6 mg/kg did not significantly affect sweat rate during exercise, nor did ingestion of caffeine in combination with water or a carbohydrate-electrolytes solution. In addition, heat dissipation was not negatively affected [93]. Therefore, while there may be an argument for caffeine-induced dieresis at rest, the literature does not indicate any significant negative effect of caffeine on sweat loss and thus fluid balance during exercise that would adversely affect performance.

## Caffeine and Doping

It has been shown that caffeine supplementation in the range of 3-6 mg/kg can significantly enhance both endurance and high-intensity performance in trained athletes. Consequently, the International Olympic Committee mandates an allowable limit of 12 μg of caffeine per ml of urine [6,15]. A caffeine dose in the range of 9 - 13 mg/kg approximately one hour prior to performance will reach the maximum allowable urinary concentration for competition [6]. Caffeine consumption and urinary concentration is dependent on factors such as gender and body weight [94]. Therefore, consuming 6-8 cups of brewed coffee that contain approximately 100 mg per cup would result in the maximum allowable urinary concentration [15,94]. According to The National Collegiate Athletic Association, urinary concentrations after competition that exceed 15 μg/ml are considered to be illegal [95]. In addition, the World Anti-Doping Agency does not deem caffeine to be a banned substance [96], but has instead included it as part of the monitoring program [97] which serves to establish patterns of misuse in athletic competition.

## Conclusion

The scientific literature associated with caffeine supplementation is extensive. It is evident that caffeine is indeed ergogenic to sport performance but is specific to condition of the athlete as well as intensity, duration, and mode of exercise. Therefore, after reviewing the available literature, the following conclusions can be drawn:

• Caffeine is more powerful when consumed in an anhydrous state (capsule/tablet, powder), as compared to coffee.

• The majority of research has utilized a protocol where caffeine is ingested 60 min prior to performance to ensure optimal absorption; however, it has also been shown that caffeine can enhance performance when consumed 15-30 min prior to exercise.

• Caffeine is effective for enhancing various types of performance when consumed in low-to-moderate doses (~3-6 mg/kg); moreover, there is no further benefit when consumed at higher dosages (≥ 9 mg/kg).

• During periods of sleep deprivation, caffeine can act to enhance alertness and vigilance, which has been shown to be an effective aid for special operations military personnel, as well as athletes during times of exhaustive exercise that requires sustained focus.

• Caffeine is an effective ergogenic aid for sustained maximal endurance activity, and has also been shown to be very effective for enhancing time trial performance.

• Recently, it has been demonstrated that caffeine can enhance, not inhibit, glycogen resynthesis during the recovery phase of exercise.

• Caffeine is beneficial for high-intensity exercise of prolonged duration (including team sports such as soccer, field hockey, rowing, etc.), but the enhancement in performance is specific to conditioned athletes.

• The literature is inconsistent when applied to strength and power activities or sports. It is not clear whether the discrepancies in results are due to differences in training protocols, training or fitness level of the subjects, etc. Nonetheless, more studies are needed to establish the effects of caffeine vis a vis strength-power sports.

• Research pertaining exclusively to women is limited; however, recent studies have shown a benefit for conditioned strength-power female athletes and a moderate increase in performance for recreationally active women.

• The scientific literature does not support caffeine-induced dieresis during exercise. In fact, several studies have failed to show any change in sweat rate, total water loss, or negative change in fluid balance that would adversely affect performance, even under conditions of heat stress.

## Competing interests

The authors declare that they have no competing interests.

## Authors' contributions

All authors read and extensively reviewed and contributed to the final manuscript.
